# Anakinra for the treatment of COVID-19 patients: a systematic review and meta-analysis

**DOI:** 10.1186/s40001-023-01072-z

**Published:** 2023-02-25

**Authors:** Karolina Dahms, Agata Mikolajewska, Kelly Ansems, Maria-Inti Metzendorf, Carina Benstoem, Miriam Stegemann

**Affiliations:** 1 Department of Intensive Care Medicine and Intermediate Care, Medical Faculty, RWTH Aachen University, Aachen, Germany; 2 Robert Koch Institute, Centre for Biological Threats and Special Pathogens (ZBS), Clinical Management and Infection Control, Berlin, Germany; 3 Institute of General Practice, Medical Faculty of the Heinrich-Heine-University Düsseldorf, Düsseldorf, Germany; 4 Department of Infectious Diseases and Respiratory Medicine, Charité – Universitätsmedizin Berlin, Corporate member of Freie Universität Berlin and Humboldt-Universität Zu Berlin, Berlin, Germany

**Keywords:** Anakinra, Interleukin 1 receptor antagonist protein, SARS-CoV-2, Hospitalization, Meta-analysis, Systematic review, COVID-19

## Abstract

**Background:**

At the end of 2021, the European Medicines Agency (EMA) expanded its approval for the recombinant human interleukin-1 (IL-1) receptor antagonist Anakinra for the treatment of COVID-19 patients with elevated soluble urokinase plasminogen activator receptor (suPAR). However, the role of Anakinra in COVID-19 remains unanswered, especially in patients receiving different forms of respiratory support. Therefore, the objective of this systematic review is to assess the safety and effects of Anakinra compared to placebo or standard care alone on clinical outcomes in adult hospitalized patients with SARS-CoV-2 infection.

**Methods:**

We searched the Cochrane COVID-19 Study Register (comprising MEDLINE, Embase, ClinicalTrials.gov, WHO International Clinical Trials Registry Platform, medRxiv, and the Cochrane Central Register of Controlled Trials (CCSR)) and the WHO COVID-19 Global literature on coronavirus disease database to identify completed and ongoing studies from inception of each database to December 13, 2021. Since then, we monitored new published studies weekly up to June 30, 2022 using the CCSR. We included RCTs comparing treatment with Anakinra to placebo or standard care alone in adult hospitalized patients with SARS-CoV-2 infection.

**Results:**

We included five RCTs with 1,627 patients (*n*_Anakinra_ = 888, *n*_control_ = 739, mean age 59.63 years, 64% male). Random-effects meta-analysis was used to pool data. We found that Anakinra makes little or no difference to all-cause mortality at up to day 28 compared to placebo or standard care alone (RR 0.96*,* 95% CI 0.64–1.45; RD 9 fewer per 1000, 95% CI 84 fewer to 104 more; 4 studies, 1593 participants; *I*^2^ = 49%; low certainty of evidence).

**Conclusions:**

Anakinra has no effect on adult hospitalized patients with SARS-CoV-2 infection regarding mortality, clinical improvement and worsening as well as on safety outcomes compared to placebo or standard care alone.

*Trial Registration*: PROSPERO Registration Number: CRD42021257552.

**Supplementary Information:**

The online version contains supplementary material available at 10.1186/s40001-023-01072-z.

## Background

Despite intensive international efforts to contain its spread and unprecedented record-speed vaccine rollout and distribution, SARS-CoV-2 has resulted in a continuously rising number of confirmed cases and deaths [1, 2], causing severe impact on healthcare facilities, healthcare workers and medical equipment.

Currently, effective treatment options are still sparse [3]. For prophylaxis and treatment, excessive immunological processes play a crucial role. Until today, there is a need for an effective anti-inflammatory and immunomodulatory therapy in COVID-19 patients. As the evidence on many of the substances that were investigated during the pandemic increased, national and international guidelines emerged to support daily clinical decisions [4–6]. However, only a few substances with proven benefits for the treatment of COVID-19 exist, such as systemic corticosteroids, interleukin-6 receptor antagonists or Janus kinase inhibitors [3].

At the end of December 2021, the European Medicines Agency (EMA) expanded its approval for the recombinant human interleukin-1 (IL-1) receptor antagonist Anakinra (r-metHuIL-1ra). Anakinra received approval for the treatment of COVID-19 patients with elevated soluble urokinase plasminogen activator receptor (suPAR). Anakinra was already approved for the treatment of certain rheumathologic diseases, i.e., rheumatoid arthritis (RA) and Still’s Disease [7]. IL-1 is a pro-inflammatory cytokine that is dysregulated in patients with severe SARS-CoV-2 infection and associated with clinical progression in COVID-19 [8]. In addition, epithelial damage by SARS-CoV-2 with a release of IL-1 β is discussed, which leads to the release of more IL-1 to recruit and activate additional innate immune cells. By competitively inhibiting the binding of cytokines to interleukin receptor antagonists, Anakinra has the potential to control active inflammation and can potentially interrupt the autoinflammatory loop [9]. Despite the approval for patients with elevated suPAR, based on data from one randomized clinical trial [10], the role and therapeutic potential of IL-1 inhibition in COVID-19 remains unanswered, especially in patients receiving different forms of respiratory support, in combination or even in comparison with other immunomodulatory substances.

The objective of this systematic review and meta-analysis is to assess the safety and effects of Anakinra compared to placebo or standard care alone on clinical outcomes in adult hospitalized patients with SARS-CoV-2 infection.

## Methods

Differences to protocol are described in the Additional file 1. The protocol for this review was registered with PROSPERO on May 28, 2021 (CRD42021257552).

### Eligibility criteria

We included randomized controlled trials (RCTs) reported as full texts, abstract only and unpublished data. We included studies comparing treatment with Anakinra to placebo or standard care alone in adult hospitalized patients with SARS-CoV-2 infection.

### Systematic search

Our Information Specialist (M.I.M.) conducted a systematic search in the following sources from inception of each database to December 13, 2021 with no restrictions on the language of publication:

- Cochrane COVID-19 Study Register (CCSR) (www.covid-19.cochrane.org), comprising:
  - MEDLINE (PubMed), daily updates;
  - Embase, weekly updates;
  - ClinicalTrials.gov (www.clinicaltrials.gov), daily updates;
  - World Health Organization International Clinical Trials Registry Platform (ICTRP) (www.who.int/trialsearch), weekly updates;
  - medRxiv (www.medrxiv.org), weekly updates;
  - Cochrane Central Register of Controlled Trials (CENTRAL), monthly updates.
- WHO COVID-19 Global literature on coronavirus disease (https://search.bvsalud.org/global-literature-on-novel-coronavirus-2019-ncov/), comprising over 15 primary sources.

Details of our search strategy are provided in the Additional file 2. Since the date of last search, we monitored new published studies weekly up to June 30, 2022 using the CCSR. Moreover, we identified other potentially eligible studies by searching the reference lists of included studies, systematic reviews and meta-analyses. We contacted authors for missing data.

### Selection of studies

We imported citations from the systematic search into Rayyan [11]. Two authors independently screened the titles and abstracts of all potential studies (K.D., K.A.). Full-text study publications were retrieved, imported into Excel and screened by two authors independently (K.D., K.A.). Reasons for exclusion of ineligible studies were recorded (Additional file 3). Any disagreements were resolved through discussion or, if required, consultation with a third author (A.M., C.B., M.S.).

### Data extraction process

We used a customised data collection form developed in Microsoft Excel [12] to collect study data (extraction tables can be requested via E-Mail). As primary outcome, we assessed all-cause mortality (day 28, day 60, time-to-event, and up to longest follow-up) and as secondary outcomes clinical status, quality of life, serious adverse events (SAE) and adverse events (AE). Extraction of study characteristics and outcome data of included studies was conducted by one author and checked by another (K.D., A.M., K.A.). Any disagreements were resolved by discussion or by consulting a third review author if necessary. Two authors transmitted the outcome data into the Cochrane statistical software RevMan 5.3 [13], which was checked by a third author (A.M.).

### Assessment of risk of bias in included studies

Two authors independently assessed the risk of bias of included studies (K.D., K.A.) using the RoB 2 tool (beta version 7) [14]. RoB 2 addresses five domains of bias (randomisation process, deviations from intended interventions, missing outcome data, measurement of the outcome, selection of the reported results). The signalling questions recommended in the tool were used to make a judgement according to the available options. Algorithms proposed in RoB 2 were used to assign each domain and the overall risk of bias, a level of bias (low risk of bias, some concerns, high risk of bias). We resolved any disagreements by discussion or by involvement of another author (A.M., M.S., C.B.).

### Measures of treatment effect

For continuous outcomes, we recorded the mean, standard deviation and total number of participants in both groups. We performed analyses using the mean difference (MD) with 95% confidence intervals (CI), if outcomes used the same scale. For dichotomous outcomes, analysed as risk ratios (RR) with 95% CI, we recorded the number of events and the total number of participants in both intervention groups. If sufficient information was available, we extracted and reported hazard ratios (HRs) for time-to-event outcomes. We contacted authors to obtain missing numerical outcome data when possible.

### Assessment of heterogeneity

We assessed statistical heterogeneity by the visual inspection of forest plots within the Review Manager (RevMan 5) software [13] and using the Chi^2^ test with a significance level of P < 0.1. We assessed statistical heterogeneity in each meta-analysis using I^2^ statistics (I^2^ > 30% to signify moderate heterogeneity, I^2^ > 75% to signify considerable heterogeneity) [15]. If I^2^ was above 75% or if there was inconsistency among the trials in the direction or magnitude of effects (judged visually), we explored possible causes for heterogeneity and used sensitivity analysis rather than subgroup analysis. Meta-analysis was not performed if no reasons for heterogeneity could be identified. Instead, the results were presented in tables.

### Assessment of reporting biases

We searched trial registries to identify completed trials that were not published elsewhere, to minimise or determine publication bias. As no more than 10 trials were included in our meta-analyses, we did not create a funnel-plot to explore potential publication bias.

### Data synthesis

We performed meta-analyses only, if the clinical and methodological characteristics of individual studies were sufficiently homogeneous. Placebo and standard care were treated as the same intervention. We used RevMan 5.3 for all analyses [13]*.* Data entry into the RevMan software was checked by a second review author for accuracy (A.M, K.D, K.A). As we anticipated that true effects would be related, but not the same for the studies included in our review, we performed random-effects meta-analyses. For continuous outcomes, we calculated mean differences with 95% CIs. We performed analyses using the inverse variance method under a random-effects model. For binary outcomes, we performed analyses using the Mantel–Haenszel method under a random-effects model to report pooled risk ratios with 95% CI.

### Summary of findings and assessment of the certainty of the evidence

We used MAGICapp software [16] to create a summary of findings table and evaluated the certainty of the evidence (A.M, C.B, M.S, K.A, K.D) using the GRADE approach for interventions evaluated in RCTs.

## Results

### Study selection

**Fig. 1:**
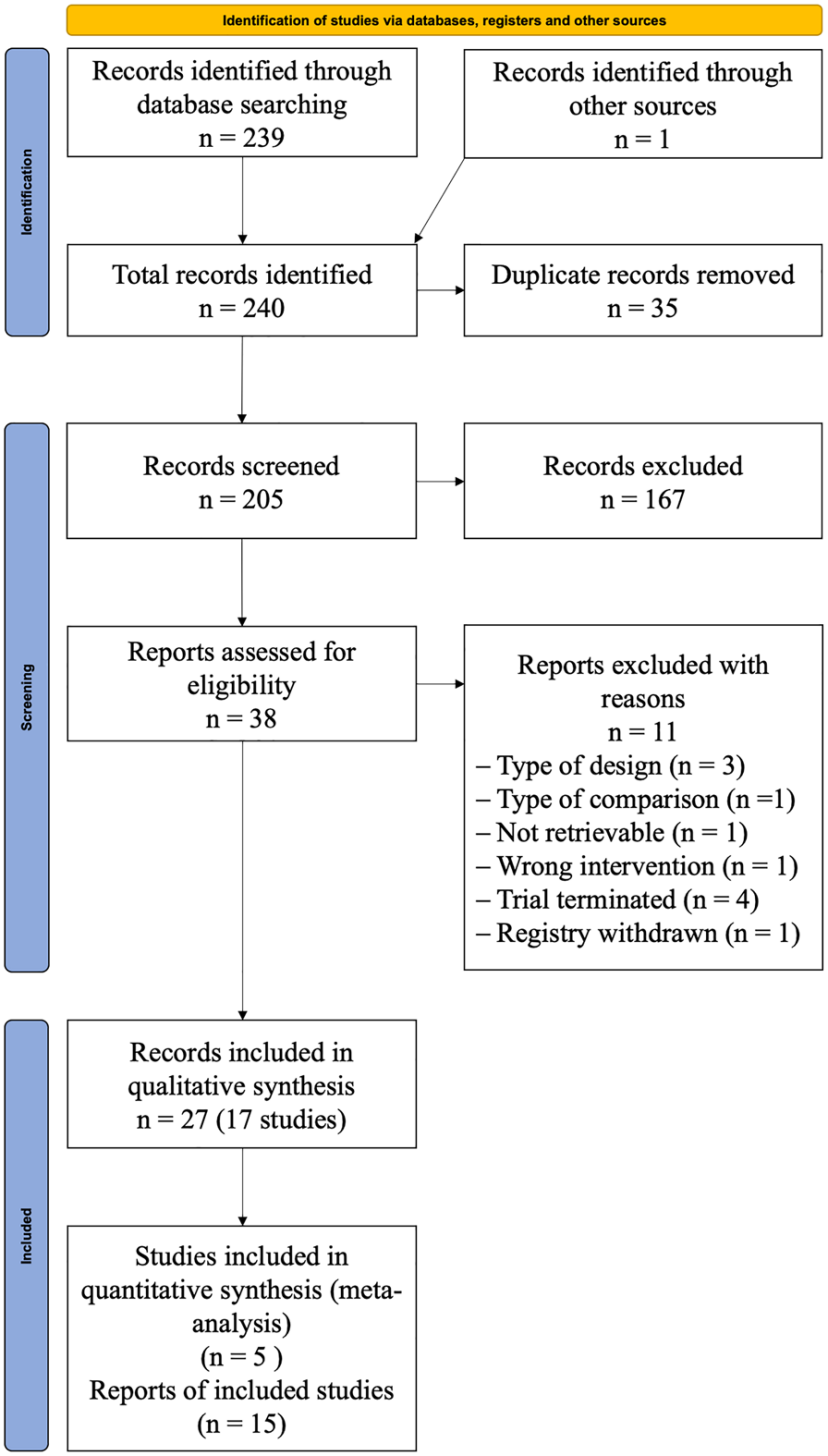
Flowchart of the systematic review selection process

The search identified 240 records. After removing duplicates, we screened 205 records based on title and abstract, of which 167 studies did not meet the prespecified inclusion criteria and were excluded. We screened the full texts and trial register entries of the remaining 38 references. Since the date of the last search, we monitored new published studies weekly until April 29, 2022 using CCSR. No additional full texts were identified. Eleven records were excluded for different reasons (Fig. 1, Additional file 3) [17–27]. The remaining 27 records were included for qualitative synthesis [10, 28–53]. Five RCTs were included in our meta-analysis: Declercq et al. [28–30], Derde et al. [31–34], Kharazmi et al. [35], Kyriazopoulou et al. [10, 36-39] and Tharaux et al. [40].

### Study characteristics

Five RCTs [10, 28, 31, 35, 40], of which one is a preprint, with a total of 1,627 adult participants (mean age 59.63 years, 64% male) diagnosed with SARS-CoV-2 infection met the criteria for inclusion and were analysed in our meta-analysis. All included RCTs used a parallel-group design. One study included only patients with elevated suPAR levels [10]. There was variability regarding setting, Anakinra dosage and duration as well as concomitant medication (Table 1). Additional study characteristics are provided in the Additional file 4.

**Table 1:** Characteristics of included studies

References	Setting; Study-design	Participants allocated, n	Age, years	Severity of condition according to respiratory support (n/N (%))	Anakinra (A) dosage	Control (C)	Concomitant medication
Declercq et al. [28]	Inpatient Multicenter in Belgium; randomized, controlled, open-label	*N*^Total^: 118 (with other interventions: 342) *n*^Anakinra^: 43^a^ *n*^Control^: 72	Median (IQR): A: 65 (54–70) C: 63 (56–73)	IMV: A: 8/43 (19); C: 9/72 (13) non-IMV: A: 16/43 (37); C: 23/72 (32) supplemental oxygen only^b^: A: 19/43 (44); C: 39/72 (54) Not requiring supplemental oxygen: A: 0/43 (0); C: 1/72 (1)	100 mg (s.c.) daily for 28 days or until hospital discharge on top of standard of care If glomerular filtration rate < 30 ml/min per 1,73 m2, the dosing was lowered to 100 mg once every other day	Standard of Care (not specified)	Antibiotics A: 21 (49%); C: 34 (47%) Glucocorticoids A: 29 (67%); C: 43 (60%) Hydroxychloroquine A: 7 (16%); C: 8 (11%) Remdesivir A: 3 (7%); C: 3 (4%)
Derde et al. [31]	Inpatient Multicenter in UK, Australia, Netherlands, Ireland, New-Zealand, Canada, Finland, Italy and Saudi Arabia; randomized, controlled, open-label	*N*^Total^: 771 (with other interventions: 2216) *n*^Anakinra^: 373 *n*^Control^: 406	Mean (SD): *Severe State* A: 59.8 (11.9) C: 61.1 (12.9) *Moderate State* A: 36.0 (17.0) C: 67.0 (13.7)	IMV: A: 138/373 (37); C: 122/406 (30) ECMO: A: 0/373 (0); C: 1/406 (0) No respiratory support/supplemental oxygen only: A: 1/373 (0); C: 2/406 (0)	300 mg (i.v.) as loading dose, followed by 100 mg every 6 h on days 1–14 or until either free from IMV for more than 24 h, or discharge from ICU If creatinine clearance < 30 ml/min or receiving renal replacement therapy, the dosing interval was increased to 12 h	Standard of Care (not specified)	Remdesivir (n (%)) A: 109 (29.5), C: 105 (26.1) Steroids (n (%)) A: 317 (85.9), C: 269 (66.9%)
Kharazmi et al. [35]	Inpatient One center in Iran; randomized, controlled, open-label	*N*^Total^: 30 *n*^Anakinra^: 15 *n*^Control^: 15	Mean (SD): A: 49.25 (19.12) C: 59.00 (1.79)	IMV or ECMO: A: 2/15 (13); C: 3/15 (20) non-IMV or high flow oxygen: A: 10/15 (67); C: 6/15 (40) low flow supplemental oxygen only: A: 3/15 (20); C: 6/15 (40)	100 mg (i.v.) daily until discharge or a maximum of 14 days	Standard of Care (not specified)	Corticosteroid A: 11 (73,3%); C: 8 (53,3%) Favipiravir A: 9 (60%); C: 4 (26,67%) Interferon A: 14 (93,3%); C: 9 (60%) Lopinavir/ritonavir A: 7 (46,67%); C: 12 (80%) Remdesivir A: 2 (13,33%); C: 4 (26,67%)
Kyriazopoulou et al [10]	Inpatient Multicenter in Greece and Italy; randomized, double-blinded, placebo-controlled	*N*^Total^: 594 *n*^Anakinra^: 405 *n*^Control^: 189	Mean (SD): A: 62 (11.4) C: 61.5 (11.3)	no supplemental oxygen: A: 39/405 (10); C: 11/189 (6) low or high flow supplemental oxygen: A: 366/405 (90); C: 178/189 (94) suPAR ≥ 6 ng/ml	100 mg (s.c.) daily for 7–10 days	Placebo: 0.9% sodium chloride daily for 7–10 days	Any glycopeptide A: 24 (5.9%); C: 19 (10.1%) Azithromycin A: 76 (18.8%); C: 35 (18.5%) β-lactamase inhibitors A: 23 (5.7%); C: 10 (5.3%) Ceftaroline A: 75 (18.5%); C: 32 (16.9%) Ceftriaxone A: 155 (38.3%); C: 85 (45.0%) Dexamethasone enrollment A: 342 (84.4%); C: 168 (88.9%) Linezolid A: 45 (11.1%); C: 22 (11.6%) Low-molecular-weight heparin A: 385 (95.1%); C: 175 (92.6%) Piperacillin/tazobactam A: 64 (15.8%); C: 36 (19.0%) Remdesivir A: 298 (73.6%); C: 141 (74.6%)Respiratory fluoroquinolone A: 53 (13.1%); C: 24 (12.7%)
Tharaux [40]	Inpatient Multicenter in France; randomized, controlled, open-label	*N*^Total^ = 114 *n*^Anakinra^ = 59 *n*^Control^ = 55	Median (IQR): A: 67 (55.5–74.3) C: 64,9 (59.5–78.3)	Low flow supplemental oxygen A: 59/59 (100) C: 55/55 (100)	2 × 200 mg (i.v.) daily on days 1–3, followed by 2 × 100 mg (i.v.) daily on day 4 and 100 mg (i.v.)/daily on day 5 In the absence of improvement (reduction in oxygen requirement by > 50%) after 3 days, decision by practitioner: 2 × 200 mg(i.v.) daily d4–6, then 2 × 100 mg (i.v.) d7, then 1 × 100 mg (i.v.) d8	Standard of Care: Antibiotics, antiviral meds, corticosteroids, vasopressors, anticoagulants (practitioner's choice)	Anticoagulants A: 33 (59%); C: 29 (53%) Azithromycin A: 11 (19%); C: 14 (25%) Dexamethasone A: 1 (2%); C: 0 (0%) Hydroxychloroquine A: 2 (3%); C: 4 (7%) Lopinavir–ritonavir or lopinavir A: 1 (2%); C: 2 (4%) Other glucocorticoids A: 6 (10%); C: 8 (15%)

^a^Characteristics of the patients at baseline available for A: 43, C: 72; outcomes partially available for A: 44, C: 74

^b^Without differentiation between low and high flow

*IMV* invasive mechanical ventilation, *s.c.* subcutaneously, *i.v.* intravenously, *ICU* intensive care unit, *ECMO* Extracorporeal membrane oxygenation, *suPAR* soluble urokinase plasminogen activator receptor

Between November 26, 2021 and February 17, 2022, we contacted the five corresponding authors to obtain missing data. Eventually, one author provided additional study characteristics and outcome data [28].

### Risk of bias assessment

The overall risk of bias among the five RCTs was low or some concerns for most outcomes due to lack of blinding among clinicians and outcome assessors as well as baseline differences (Additional file 5).

### Effects of interventions

Anakinra compared to placebo or standard care alone (Table 2).

**Table 2:** Summary of findings

Anakinra compared to placebo or standard care alone on clinical outcomes in SARS-CoV-2 patients					
Outcomes Time frame of absolute effects	Absolute effects from study(ies) (95% CI)			Relative effect 95% CI	Quality of the evidence (GRADE)
	Placebo or Standard Care Alone	Anakinra	Difference with Anakinra		
28-day mortality	232 per 1000	223 per 1000 (148 to 336)	9 Fewer per 1000 (84 Fewer—104 More); 4 studies; 1593 participants	0.96 (0.64 to 1.45)	⊕ ⊕ ⊖ ⊖ LOW Due to serious inconsistency, Due to serious imprecision
60-day mortality	125 per 1000	233 per 1000 (102 to 526)	108 More per 1000 (23 Fewer—401 More); 1 study; 115 participants	1.86 (0.82 to 4.21)	⊕ ⊕ ⊖ ⊖ LOW Due to very serious imprecision
Mortality at hospital discharge	331 per 1000	404 per 1000 (254 to 635)	73 More per 1000 (76 Fewer—305 More); 2 studies; 889 participants	1.22 (0.77 to 1.92)	⊕ ⊕ ⊕ ⊖ MODERATE Due to serious imprecision
Clinical worsening: new need for invasive mechanical ventilation or death (at day 28)	138 per 1000	95 per 1000 (42 to 215)	43 Fewer per 1000 (95 Fewer—77 More); 2 studies; 709 participants	0.69 (0.31 to 1.56)	⊕ ⊕ ⊖ ⊖ LOW Due to serious inconsistency, Due to serious imprecision
Clinical worsening: new need for invasive mechanical ventilation or death (at day 28) in patients with suPAR ≥ 6 ng/ml	127 per 1000	62 per 1000 (36 to 105)	65 Fewer per 1000 (90 Fewer—22 Fewer); 1 study; 594 participants	0.49 (0.29 to 0.83)	⊕ ⊕ ⊕ ⊖ MODERATE Due to serious imprecision
Clinical improvement: discharged without clinical deterioration (at day 28)	744 per 1000	766 per 1000 (654 to 900)	22 More per 1000 (89 Fewer—156 More); 3 studies; 823 participants	1.03 (0.88 to 1.21)	⊕ ⊕ ⊖ ⊖ LOW Due to serious inconsistency, Due to serious imprecision
Serious adverse events	241 per 1000	246 per 1000 (163 to 368)	5 More per 1000 (77 Fewer—128 More); 3 studies, 823 participants	1.02 (0.68 to 1.53)	⊕ ⊕ ⊖ ⊖ LOW Due to serious inconsistency, Due to serious imprecision
Adverse events (any grade)	520 per 1000	556 per 1000 (436 to 712)	36 More per 1000 (83 Fewer—192 More); 2 studies; 229 participants	1.07 (0.84 to 1.37)	⊕ ⊕ ⊖ ⊖ LOW Due to serious risk of bias, Due to serious imprecision
Adverse events (grades 3–4)	333 per 1000	373 per 1000 (223 to 616)	40 More per 1000 (110 Fewer—283 More); 1 study; 115 participants	1.12 (0.67 to 1.85)	⊕ ⊖ ⊖ ⊖ VERY LOW Due to serious risk of bias, Due to very serious imprecision

### All-cause mortality

**Fig. 2:**
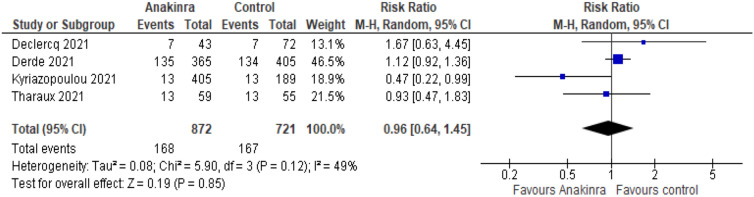
Forest plot describing the difference between Anakinra compared to placebo or standard care alone regarding all-cause mortality at up to day 28. *M–H* Mantel–Haenszel

Four studies reported all-cause mortality at up to day 28 for 1593 participants (Fig. 2). We found that Anakinra makes little or no difference to all-cause mortality at up to day 28 compared to placebo or standard care alone (RR 0.96*,* 95% CI 0.64–1.45; risk difference (RD) 9 fewer per 1000, 95% CI 84 fewer to 104 more; 4 studies, 1593 participants; *I*^2^ = 49%; low certainty of evidence). The reason for downgrading was serious inconsistency due to inconsistent direction and widely varying point estimates and serious imprecision due to wide CIs and that the CI includes both benefits and harms.

Analyses regarding all-cause mortality at up to 60 days, time-to-event and at hospital discharge are reported in the supplements (Additional file 6: Figs. S1–S4).

### Worsening of clinical status

#### New need for invasive mechanical ventilation or death

**Fig. 3:**
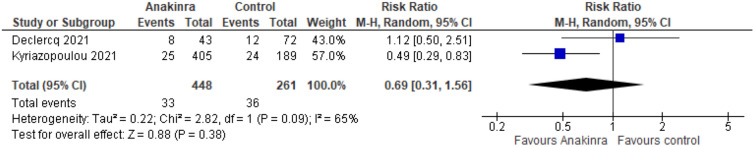
Forest plot describing the difference between Anakinra compared to placebo or standard care alone regarding worsening of clinical status. *M–H* Mantel–Haenszel

Two studies reported this outcome at day 28 for 709 participants (Fig. 3). We found that treatment with Anakinra has no effect on the risk for invasive mechanical ventilation (RR 0.69, 95% CI 0.31–1.56; RD 43 fewer per 1000, 95% CI 95 fewer to 77 more; 2 studies, 709 participants; *I*^2^ = 65%; low certainty of evidence). Our main reason for downgrading was serious inconsistency due to statistical heterogeneity, inconsistent direction and widely varying point estimates and serious imprecision due to wide CIs and that the CI includes both benefits and harms.

One study reported this outcome at day 28 in 594 patients with increased suPAR levels (Fig. 3, Kyriazopoulou and Additional file 6: Fig. S5). We found that treatment with Anakinra probably decreases the risk for a new need for invasive mechanical ventilation or death (RR 0.49, 95% CI 0.29–0.83; RD 65 fewer per 1000, 95% CI 90 fewer to 22 more; 1 study, 594 participants; I^2^ not applicable; moderate certainty of evidence). Our main reason for downgrading was serious imprecision due to data from only one study.

### Improvement of clinical status

#### Discharged without clinical deterioration

**Fig. 4:**
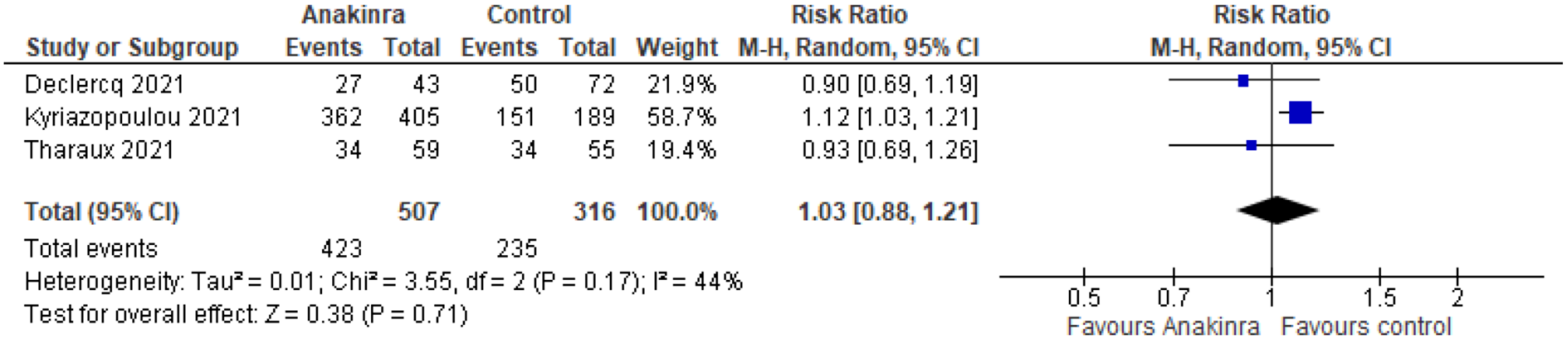
Forest plot describing the difference between Anakinra compared to placebo or standard care alone regarding improvement of clinical status. *M–H* Mantel–Haenszel

Three studies reported this outcome at day 28 for 823 participants (Fig. 4). We found that treatment with Anakinra has no effect on being discharged without clinical deterioration at day 28 (RR 1.03, 95% CI 0.88–1.21; RD 22 more per 1000, 95% CI 89 fewer to 156 more; 3 studies, 823 participants; *I*^2^ = 44%; low certainty of evidence). Our reasons for downgrading were serious inconsistency due to inconsistent direction and widely varying point estimates and serious imprecision due to wide CIs and that the CI includes both benefits and harms.

### Quality of life

We did not find any data for this outcome.

### Safety outcomes

#### Serious adverse events

Three studies reported this outcome for 823 participants (Additional file 6: Fig. S6). We found that treatment with Anakinra probably has little or no difference on SAE compared to standard care plus/minus placebo (RR 1.02, 95% CI 0.68–1.53; RD 5 more per 1000, 95% CI 77 fewer to 128 more; 3 studies, 823 participants; *I*^2^ = 56%; low certainty of evidence). The reasons for downgrading were serious inconsistency due to inconsistent direction and widely varying point estimates and serious imprecision due to wide CIs and that CI includes both benefits and harms.

#### Adverse events

Two studies reported any AE for 229 participants (Additional file 6: Fig. S7). We found that treatment with Anakinra may have little or no difference on AE (RR 1.18, 95% CI 0.78–1.76; RD 36 more per 1000, 95% CI 83 fewer to 192 more; two studies, 229 participants; *I*^2^ = 0%; low certainty of evidence). The reasons for downgrading were serious risk of bias due to lack of blinding and serious imprecision due to wide CIs and the fact that CI includes both benefits and harms.

One study reported AE grades 3–4 for 115 participants (Additional file 6: Fig. S8). We found that treatment with Anakinra may have little or no difference on AE (RR 1.12, 95% CI 0.67–1.85; RD 40 more per 1000, 95% CI 110 fewer to 283 more; one study, 115 participants; I^2^ not applicable; very low certainty of evidence). The main reason for downgrading was serious risk of bias due to lack of blinding and very serious imprecision due to wide CIs, few patients and data from only one study.

## Discussion

Five RCTs comparing Anakinra treatment with placebo or standard care alone in 1,627 hospitalized patients with SARS-CoV-2 infection were included. There was variability regarding setting, Anakinra dosage and duration as well as concomitant medication.

Regarding the primary outcome, we found that Anakinra makes little or no difference to all-cause mortality at up to day 28 compared to placebo or standard care alone. Regarding secondary outcomes, the meta-analysis showed no benefit for Anakinra with respect to clinical status, quality of life, SAEs and AEs.

The Cochrane review of Davidson et al. [55], which examined two RCTs [10, 40] for all-cause mortality, came to a similar conclusion as our meta-analysis. In addition, our findings regarding clinical improvement are also supported by Davidson et al. [55]. Regarding the outcome clinical worsening, neither of us found an effect of Anakinra, but because different studies were included in the meta-analyses, the results are only comparable to a limited extent in terms of content. Davidson et al. [55] performed meta-analysis with the studies of Kyriazopoulou et al. [10] and Tharaux et al. [40], while we included the studies of Kyriazopoulou et al. [10] and Declercq et al. [28] in our meta-analysis.

Contrary to our review, a meta-analysis published in Lancet Rheumatology showed a significant reduction in mortality in patients with moderate to severe COVID-19 receiving Anakinra compared to standard of care with or without placebo [54]. However, this review included mostly observational studies and only one RCT. The systematic review of Somagutta et al. [56], which analysed severe cases of COVID-19, concluded that the use of Anakinra for patients with COVID-19 was associated with a significantly low mortality rate and mechanical ventilation compared with standard care alone. Though, this review included only one RCT and mainly observational studies, case series and case reports.

Regarding clinical improvement by day 28, our analysis detected a favourable effect of Anakinra when considering only one study using biomarker-guided therapy (suPAR ≥ 6 ng/ml) [10]. As this biomarker was not determined in any other study, a comparison to other study data as well as to a subgroup with non-elevated suPAR is not possible. Thus, the role of the elevated suPAR biomarker as a determinant of response to Anakinra therapy is uncertain. Despite this result, current treatment recommendations [9] are often based on the beneficial effect reported by Kyriazopoulou et al. [10]. Therefore, it is of clinical importance to investigate the role of suPAR as biomarker for the treatment of COVID-19 with Anakinra.

Our search for systematic reviews and meta-analysis showed that most systematic reviews analysing the effect of Anakinra in adult hospitalized COVID-19 patients include mainly observational studies [54, 56–63]. However, to make evidence-based recommendations, it is of great interest that RCTs are performed because of their higher level of evidence.

## Limitations

The outcomes of interest were revised and partially changed compared to the protocol, due to new knowledge regarding their clinical relevance. Moreover, one of the included studies is a preprint, which has not yet been peer-reviewed and could change until publication. Due to the selected inclusion criteria only few studies were included. Furthermore, the approach of each study regarding their inclusion criteria was heterogeneous. Therefore, meta-analysis was not always possible. In addition, the analyses conducted compare results of studies that differ regarding their patient population, especially disease severity, degree of oxygenation impairment, biomarkers, concomitant medication, Anakinra dosage studied and duration of therapy. However, subgroup analysis was not possible, due to the fact that the data needed for analyses were not available.

## Conclusion

Anakinra has no effect on adult hospitalized patients with SARS-CoV-2 infection regarding mortality, clinical improvement or worsening as well as on safety outcomes compared to placebo or standard care alone. However, there might be a potential benefit of therapy with Anakinra in hospitalized patients with COVID-19 with low-flow/high-flow oxygen therapy and suPAR ≥ 6 ng/ml regarding the need for invasive mechanical ventilation or death by day 28.

## Supplementary Information

**Additional file 1.** Differences to protocol.

**Additional file 2.** Search strategies.

**Additional file 3.** Records excluded during full-text screening.

**Additional file 4.** Characteristics of included studies.

**Additional file 5.** Risk of bias assessment.

**Additional file 6.** Meta-analyses of Anakinra versus control.

## Data Availability

All data generated or analysed during this review are included in this published article and its supplementary information files.

## Abbreviations

ZBS: Centre for Biological Threats and Special Pathogens
CCSR: Cochrane Central Register of Controlled Trials
RCT: Randomized controlled trials
EMA: European Medicines Agency
IL-1: Interleukin-1 receptor antagonist
suPAR: Soluble urokinase plasminogen activator receptor
RA: Rheumatoid arthritis
ICTRP: International clinical trials registry platform
SAE: Serious adverse events
AE: Adverse events
MD: Mean difference
CI: Confidence interval
RR: Risk ratio
HR: Hazard ratio
IMV: Invasive mechanical ventilation
ICU: Intensive care unit
i.v.: Intravenously
s.c.: Subcutaneously
ECMO: Extracorporeal membrane oxygenation
RD: Risk difference
