# Supplementary material for: Anakinra for the treatment of COVID-19 patients: a systematic review and meta-analysis
Source: Eur J Med Res. 2023 Feb 25;28:100. doi: 10.1186/s40001-023-01072-z (PMC9959952; doi:10.1186/s40001-023-01072-z)
Supplement: Supplementary file 1 — Additional file 1. Differences to protocol. [file 40001_2023_1072_MOESM1_ESM.pdf]

Supplementary Information No 1 Differences to protocol

1. Due to the fact that 'no treatment' is only applicable within the ambulatory setting, we chose to change the review question into the following: The objective of this systematic review is to assess the safety and effects of Anakinra compared to placebo or standard care alone on clinical outcomes in adult hospitalized patients with SARS-CoV-2 infection.
2. Due to changes of outcomes within the CEOsys project, the outcome set was changed on the basis of clinical relevance. The following outcomes were used in the current publication:

Prioritised outcomes

- All-cause mortality at day 28, day 60, time-to-event, and up to longest follow-up.
- Clinical status at day 28, day 60, and up to longest follow-up, including:
  - worsening of clinical status:
    - participants with clinical deterioration (new need for invasive mechanical ventilation) or death;
  - improvement of clinical status:
    - participants discharged alive. Participants should be discharged without clinical deterioration or death.
- Quality of life, including fatigue and neurological status, assessed with standardised scales (e.g. WHOQOL-100) at up to seven days; up to 30 days, and longest follow-up available.

Safety outcomes

- Serious adverse events during the study period, defined as number of participants with any serious adverse event (serious as defined according to CTCAE (Common Terminology Criteria for Adverse Events)).
- Adverse events (any grade, grade 3-4) during the study period, defined as the number of participants with any adverse event.
