# Supplementary material for: Anakinra for the treatment of COVID-19 patients: a systematic review and meta-analysis
Source: Eur J Med Res. 2023 Feb 25;28:100. doi: 10.1186/s40001-023-01072-z (PMC9959952; doi:10.1186/s40001-023-01072-z)
Supplement: Supplementary file 2 — Additional file 2. Search strategies. [file 40001_2023_1072_MOESM2_ESM.pdf]

Supplementary Information No 2 Search strategies

Cochrane COVID-19 Study Register

Search string:

Anakinra OR "IL1 Febrile Inhibitor" OR "Interleukin 1 Inhibitor" OR Antril OR Kineret OR "Interleukin 1 Receptor Antagonist" OR "IL-1Ra" OR "IL-1 Inhibitor" OR "IL 1Ra" OR "IL 1 Inhibitor"

Study characteristics:

1) "Intervention assignment": “Randomised” OR “unclear” OR

2) "Study type": "Interventional" AND "Study design": "Parallel/Crossover"

3) "Study type": "Interventional" AND "Study design": "Unclear"

4) "Study type": "Adaptive/Platform"

= 92 references

WHO COVID-19 Global literature on coronavirus disease

Title, abstract, subject:

(Anakinra OR "IL1 Febrile Inhibitor" OR "Interleukin 1 Inhibitor" OR Antril OR Kineret OR "Interleukin 1 Receptor Antagonist" OR "IL-1Ra" OR "IL-1 Inhibitor" OR "IL 1Ra" OR "IL 1 Inhibitor") AND (random* OR placebo OR trial OR groups OR "phase 3" or "phase3" or p3 or "pIII")

= 147 references
