# Supplementary material for: Anakinra for the treatment of COVID-19 patients: a systematic review and meta-analysis
Source: Eur J Med Res. 2023 Feb 25;28:100. doi: 10.1186/s40001-023-01072-z (PMC9959952; doi:10.1186/s40001-023-01072-z)
Supplement: Supplementary file 3 — Additional file 3. Records excluded during full-text screening. [file 40001_2023_1072_MOESM3_ESM.pdf]

Supplementary Information No 3 Records excluded during full-text screening

**Author**	**Year**	**Title**	**Type**	**Reason for exclusion in full-text screening**
Assistance Publique Hopitaux De Marseille<br>NCT04424056	2020	A Trial Using ANAKINRA, TOCILIZUMAB Alone or in Association With RUXOLITINIB in Severe Stage 2b and 3 of COVID19-associated Disease	Trial registry	Wrong intervention
Kyriazopoulou et al.<br>Preprint	2020	Anakinra To Prevent Respiratory Failure In COVID-19	Preprint	Wrong study design
Kyriazopoulou et al.	2021	An open label trial of anakinra to prevent respiratory failure in COVID-19	Journal publication	Wrong study design
University of Manchester<br>EUCTR2020-001636-95-GB	2020	Does subcutaneously(SC) administered of anakinra achieve good penetration in blood and result in reduce inflammation in patients with SARS-CoV-19?	Trial registry	Wrong comparator
Swedish Orphan Biovitrum<br>EUCTR2020-001167-93	2020	A clinical trial investigating efficacy and safety of emapalumab and anakinra in patients with Coronavirus disease (COVID-19)	Trial registry	Wrong comparator
Kaplanski G, Bontemps D, Esnault P, et al.	2020	Combined Anakinra and Ruxolitinib treatment to rescue extremely ill COVID-19 patients: a pilot study	Journal publication	Wrong comparator
Swedish Orphan Biovitrum<br>NCT04324021	2020	Efficacy and Safety of Emapalumab and Anakinra in Reducing Hyperinflammation and Respiratory Distress in Patients With COVID-19 Infection	Trial registry	Trial terminated

University Hospital Tours<br><br>NCT04364009	2020	Anakinra for COVID-19 Respiratory Symptoms	Trial registry	Trial terminated
Centre Hospitalier Intercommunal de Toulon La Seyne sur Mer<br><br>NCT04366232	2020	Efficacy of Intravenous Anakinra and Ruxolitinib During COVID-19 Inflammation (JAKINCOV)	Trial registry	Trial terminated
CHRU de TOURS<br><br>EUCTR2020-001734-36	2020	Efficacy and safety of ANAkinra during Adult COVID-19 with Aggravating respiratory symptoms: a multicenter open-label controlled randomized trial	Trial registry	Trial terminated
Weill Medical College of Cornell University<br><br>NCT04603742	2020	Anakinra, COVID-19, Cytokine Storm	Trial registry	Registry withdrawn
