# Supplementary material for: Anakinra for the treatment of COVID-19 patients: a systematic review and meta-analysis
Source: Eur J Med Res. 2023 Feb 25;28:100. doi: 10.1186/s40001-023-01072-z (PMC9959952; doi:10.1186/s40001-023-01072-z)
Supplement: Supplementary file 4 — Additional file 4. Characteristics of included studies. [file 40001_2023_1072_MOESM4_ESM.pdf]

Supplementary Information No 4 Characteristics of included studies

Declercq 2021
Declercq J, van Damme KFA, De Leeuw E, et al: Effect of anti-interleukin drugs in patients with COVID-19 and signs of cytokine release syndrome (COV-AID): a factorial, randomised, controlled trial. *Lancet Respir Med* 2021; 9:1427–38

**Design**	**Study period and place**	**Participants**	**Intervention**	**Control**	**Outcomes**	**Conclusion**
Randomized controlled trial,<br>2x2 factorial design,<br>open-label,<br>Inpatient	16 hospitals in Belgium	**Patients:**<br>N^{Total}: 118 (with other interventions: 342)<br>A: n = 43[1]<br>C: n = 72<br>Excluded:<br>Not reported for Anakinra and control only<br><br><u>Follow-up:</u><br>10-20 weeks after randomization<br><br>**Inclusion criteria:**<br>− Recent infection with COVID-19<br>− Presence of hypoxia<br>− Signs of cytokine release syndrome<br>− Chest x-ray and/or CT scan showing bilateral infiltrates within last 2 days<br>− Admitted to specialized COVID-19 ward or an ICU ward taking care of COVID-19 patients<br>− ≥ 18 years<br>− Male or female<br>− Women of childbearing potential must have a negative serum pregnancy test pre-dose on day 1<br>− Willing and able to provide informed consent or legal representative willing to provide informed consent<br><br>**Exclusion criteria:**<br>− Patients with known history of serious allergic reactions, including anaphylaxis to any of the study medications, or any component of the product<br>− Mechanical ventilation > 24h at randomization<br>− Patient on ECMO at time of screening<br>− Clinical frailty scale above 3<br>− Active bacterial or fungal infection<br>− Unlikely to survive beyond 48 h<br>− Neutrophil count below 1500 cells/microliter<br>− Platelets below 50.000/microliter<br>− Patients enrolled in another investigational drug study	**Anakinra**<br>1x/day 100mg subcutaneously for 28 days or until hospital discharge on top of standard of care<br><br>If glomerular filtration rate <30 ml/min per 1,73 m2, the dosing was lowered to 100mg once every other day.	**Standard of Care** (not specified)	**Primary outcome:**<br><u>Time to clinical improvement</u><br>Number of deaths<br>A: 10/44 (23%)<br>C: 9/74 (12%)<br><br>**Secondary outcomes:**<br>− Median time until discharge (IQR)<br>A: 14 (9-37)<br>C: 12 (10-20)<br>− Median time until independence from supplemental oxygen or discharge<br>A: 14 (8-20)<br>C: 11 (10-15)<br>− Median time until independence from invasive ventilation<br>A: <50% reached event<br>C: 54 (6-.)<br>− Median time until first use of high-flow oxygen device, ventilation or death<br>A: <50% reached event<br>C: <50% reached event<br>− Number of days in hospital<br>A: 20 (16-25)<br>C: 18 (15-22)<br>− Number of days in ICU<br>A: 12 (7-21)<br>C: 9 (6-14)	No shortening of time to clinical improvement or improvement of supportive endpoints when given early in disease course of hypoxia patients with COVID-19 with evidence of CRS.<br>No increase in infectious adverse events or other safety concerns associated with use of Anakinra.<br><br>**Comments:**<br><u>Limitations:</u><br>− Open-label design<br>− Standard of care could differ among centres and did change during the course of the trial<br>− small sample size<br>− total number of patients screened for eligibility not registered in all centres<br>− decision to exclude patients unlikely to survive beyond 48h made by the individual enrolling physician on clinical grounds without objective criteria beyond the use of a frailty scoring index

Supplementary Information No 4 Characteristics of included studies

Declercq 2021
Declercq J, van Damme KFA, De Leeuw E, et al: Effect of anti-interleukin drugs in patients with COVID-19 and signs of cytokine release syndrome (COV-AID): a factorial, randomised, controlled trial. *Lancet Respir Med* 2021; 9:1427–38

Design	Study period and place	Participants	Intervention	Control	Outcomes	Conclusion
		− Patients on high dose systemic steroids<br>− Patients on immunosuppressant or immunomodulatory drugs<br>− Patients on current anti-IL1 or anti-IL6 treatment<br>− Signs of active tuberculosis<br>− Serum transaminase levels >5times upper limit of normal, unless there are clear signs of cytokine release syndrome<br>− History of (non-iatrogenic) bowel perforation or diverticulitis<br>− Pregnant or breastfeeding females<br><br>**Demographics:**<br>Age, median (IQR):<br>A: 65 (54 - 70)<br>C: 63 (56 - 73)<br>Male, n (%)<br>A: 37 (86)<br>C: 53 (74)<br>**Severity of condition according to respiratory support (n/N (%)):**<br>IMV:<br>A: 8/43 (19); C: 9/72 (13)<br>non-IMV:<br>A: 16/43 (37); C: 23/72 (32)<br>supplemental oxygen only[2]:<br>A: 19/43 (44); C: 39/72 (54)<br>Not requiring supplemental oxygen:<br>A: 0/43 (0); C: 1/72 (1)				

[1]characteristics of the patients at baseline available for A: 43, C: 72; outcomes partially available for A: 44, C: 74
[2]without differentiation between low and high flow

Derde 2021
The REMAPCAP Investigators: Effectiveness of Tocilizumab, Sarilumab, and Anakinra for critically ill patients with COVID-19 The REMAP-CAP COVID-19 Immune Modulation Therapy Domain Randomized Clinical Trial. medRxiv [Preprint] 2021.06.18.21259133; doi: https://doi.org/10.1101/2021.06.18.21259133

Design	Study period and place	Participants	Intervention (Anakinra, A)	Control (C)	Outcomes	Conclusion of study authors
Randomized controlled trial,	19.04.2020 - 10.04.2021,	**Patients:**<br>**s**creened: n = 13,718<br>randomised to a COVID-19 Immune	**Anakinra (A)**<br>300mg IV loading dose	**Standard of Care** (not specified)	**Primary outcome[1]:**<br>In-hospital death, n (%)	“Anakinra is not effective in this population”<br><br>**Comments:**

Supplementary Information No 4 Characteristics of included studies

Declercq 2021
Declercq J, van Damme KFA, De Leeuw E, et al: Effect of anti-interleukin drugs in patients with COVID-19 and signs of cytokine release syndrome (COV-AID): a factorial, randomised, controlled trial. *Lancet Respir Med* 2021; 9:1427–38

Design	Study period and place	Participants	Intervention	Control	Outcomes	Conclusion
open-label,<br>Inpatient,<br>(Preprint)	127 sites (4 sites for Moderate state (3 UK, 1 Australia) & 123 sites for Severe State (110 UK, 11 Netherlands, 3 Ireland, 2 Australia, 2 New-Zealand, 1 Canada, 1 Finland, 1 Italy, 1 Saudi-Arabia)	Modulation Domain intervention: n=2,279<br>N= 779<br>A: n = 373<br>C: n = 406<br>Excluded:<br>A: 13<br>C: 12<br><u>Follow-up:</u><br>- 21 d for primary outcome and 90d for secondary outcomes<br><br>**Inclusion criteria:**<br>− > 18 years<br>− within 24 hours of receiving respiratory or cardiovascular organ support in an ICU<br>− suspected or microbiologically confirmed COVID-19<br><br>**Exclusion criteria:**<br>- Patient has already received any dose of one or more of any form of anakinra during this hospitalization or is on long-term therapy with this agent prior to this hospital admission<br>- Known condition or treatment resulting in ongoing immune suppression including neutropenia prior to this hospitalization<br>- Patient has been randomized in a trial evaluating an immune modulation agent for proven or suspected COVID-19 infection, where the protocol of that trial requires ongoing administration of study drug<br>- The treating clinician believes that participation in the domain would not be in the best interests of the patient<br>- Known hypersensitivity to anakinra<br>- Known hypersensitivity o proteins produced by E. coli<br>- Known or suspected pregnancy<br><br>**Demographics:**<br><u>Age, mean (SD)</u><br>Severe state<u>:</u><br>A: 59.8 (11.9)<br>C: 61.1 (12.9)	100mg every 6 hours for 14 days or until for > 24 h free from IMV or discharge from ICU.<br><br>creatinine clearance <30ml/min or receiving renal replacement therapy: dosing interval increased to 12 h		A: 145/365 (39.7)<br>C: 150/406 (36.9)<br><br>**Secondary outcomes:**<br>90-day mortality, adjusted HR – mean (SD)<br>A: 1.15 (0.16)<br>C: 1<br>Progression into intubation, ECMO or death, n (%)<br>A: 122/228 (53.5)<br>C: 147/276 (53.3)<br>Time to ICU discharge, adjusted HR – mean (SD)<br>A; 1.10 (0.12)<br>C: 1<br>Time to hospital discharge, adjusted HR – mean (SD)<br>A: 1.05 (0.12)<br>C: 1	<u>Limitations</u>:<br>− open-label design

Supplementary Information No 4 Characteristics of included studies

Declercq 2021
Declercq J, van Damme KFA, De Leeuw E, et al: Effect of anti-interleukin drugs in patients with COVID-19 and signs of cytokine release syndrome (COV-AID): a factorial, randomised, controlled trial. *Lancet Respir Med* 2021; 9:1427–38

Design	Study period and place	Participants	Intervention	Control	Outcomes	Conclusion
		Moderate State:<br>A: 36.0 (17.0)<br>C: 67.0 (13.7)<br><br><u>Male, n (%)</u><br>Severe state:<br>A: 269 (72.1)<br>C: 285 (70.2)<br>Moderate State:<br>A: 1 (50.0)<br>C: 1 (33.3)<br><br>**Severity of condition according to respiratory support (n/N (%)):**<br>None / supplemental oxygen only<br>A: 1/373 (0.3)<br>C: 2/406 (0.5)<br>High-flow nasal cannula<br>A: 101/373 (27.1)<br>C: 110/406 (27.1)<br>Non-invasive ventilation only<br>A: 133/373 (35.7)<br>C: 171/406 (42.1)<br>Invasive mechanical ventilation<br>A: 138/373 (37.0)<br>C: 122/406 (30.0)<br>ECMO<br>A: 0/373 (0.0)<br>C: 1/406 (0.2)				

[1] The primary outcome was an ordinal scale that is a composite of in-hospital mortality and duration of respiratory and cardiovascular organ support (Days free from organ support in survivors, median (IQR))

Kharazmi 2021
Kharazmi AB, Moradi O, Haghighi M, et al: A randomized controlled clinical trial on efficacy and safety of anakinra in patients with severe COVID-19. *Immun Inflamm Dis* 2022; 10:201–08

Design	Study period and place	Participants	Intervention	Control	Outcomes	Conclusion
Randomized controlled trial,<br>open-label,<br>Inpatient,	Iran,<br>May 2020 to July 2020	**Patients:**<br>Screening: n = 72<br>Patients: n = 30<br>A: n = 15<br>C: n = 15<br>Excluded: n = 42	**Anakinra**<br>1x/day 100mg IV until discharge or a maximum of 14 days	**Standard of Care** (not specified)	**Primary outcome:**<br><u>Need for invasive mechanical ventilation</u><br><br>**Secondary outcomes:**	"Anakinra is effective in improving the respiratory condition and significantly reduces the need for invasive mechanical ventilation in patients with severe COVID-19. "

Supplementary Information No 4 Characteristics of included studies

**Declercq 2021**<br>Declercq J, van Damme KFA, De Leeuw E, et al: Effect of anti-interleukin drugs in patients with COVID-19 and signs of cytokine release syndrome (COV-AID): a factorial, randomised, controlled trial. *Lancet Respir Med* 2021; 9:1427–38						
**Design**	**Study period and place**	**Participants**	**Intervention**	**Control**	**Outcomes**	**Conclusion**
		Follow-up:<br>for the primary outcome: until endotracheal intubation due to hypoxemia<br>for the secondary outcomes: until hospital discharge<br><br>**Inclusion criteria:**<br>– confirmed diagnosis of COVID-19 based on reverse transcriptase-polymerase chain reaction<br>– admitted to ICU<br>– > 18 years old<br>– elevated C-reactive protein (CRP) levels<br>– oxygen saturation ≤ 93% measured using a peripheral capillary pulse oximeter<br>– fever (core temperature of 37.8°C or more), or cough or shortness of breath, and PaO2/FiO2 less than 300<br><br>**Exclusion criteria:**<br>– Patients who had positive results for tuberculosis (i.e., positive Mendel–Mantoux or QuantiFERON test),<br>– viral hepatitis B or C<br>– hemoglobin < 7.5 g/dl<br>– platelet count < 100,000 cells/µl<br>– serum glutamic–oxaloacetic transaminase or serum glutamic–pyruvic transaminase > five upper limits of normal<br>– untreated active infection<br>– previous administration of anakinumab or anakinra<br><br>**Demographics:**<br>Age, (SD)<br>A: 49.25 ± 19.12<br>C: 59.00 ± 1.79<br><br>Male, (%)<br>A: 8%<br>C: 7%			Hospital length of stay, Median (IQR)<br>A: 10 (5)<br>C: 28 (15)<br><br>ICU length of stay, Median (IQR)<br>A: 5 (3)<br>C: 16 (19)<br><br>Seven categories ordinal scale, n (%)<br>Death<br>A: 5 (33.3)<br>C: 7 (46.7)<br>p = .456<br>Hospitalized, on invasive mechanical ventilation or extracorporeal membrane oxygenation.<br>A: 0 (0)<br>C: 2 (13.3)<br>P = .483<br>Hospitalized, on non-invasive ventilation or high flow oxygen<br>A: 0 (0)<br>C: 1 (6.7)<br>P = 1.000<br>Hospitalized, requiring low flow supplemental oxygen<br>A: 0 (0)<br>C: 0 (0)<br>P = 1.000<br>Hospitalized, not requiring supplemental oxygen—requiring ongoing medical care (COVID-19 related or otherwise)<br>A: 0 (0)<br>C: 0 (0)<br>P = 1.000	"The reduction was observed in hospitalization duration, which makes the medication an effective immunomodulatory agent to combat cytokine storm."<br><br>**Comments:**<br>Limitations:<br>– Open label design<br>– Small sample size

Supplementary Information No 4 Characteristics of included studies

Declercq 2021
Declercq J, van Damme KFA, De Leeuw E, et al: Effect of anti-interleukin drugs in patients with COVID-19 and signs of cytokine release syndrome (COV-AID): a factorial, randomised, controlled trial. *Lancet Respir Med* 2021; 9:1427–38

Design	Study period and place	Participants	Intervention	Control	Outcomes	Conclusion
		**Severity of condition according to respiratory support (n/N (%)):**<br>IMV or ECMO:<br>A: 2/15 (13); C: 3/15 (20)<br>non-IMV or high flow oxygen:<br>A: 10/15 (67); C: 6/15 (40)<br>low flow supplemental oxygen only:<br>A: 3/15 (20); C: 6/15 (40)			Hospitalized, not requiring supplemental oxygen—no longer required ongoing medical care.<br>A: 0 (0)<br>C: 0 (0)<br>P = 1.000<br>Not hospitalized<br>A: 10 (66.7)<br>C: 5 (33.3)<br>P = .143<br><br>Survival for included patients on day 14	

Kyriazopoulou 2021
Kyriazopoulou E, Poulakou G, Milionis H, et al: Early treatment of COVID-19 with anakinra guided by soluble urokinase plasminogen receptor plasma levels: a double-blind, randomized controlled phase 3 trial. *Nat Med* 2021; 27:1752–1760

Design	Study period and place	Participants	Intervention	Control	Outcomes	Conclusion
Randomized controlled trial; double-blinded, placebo-controlled, Inpatient	23 December 2020 to 31 March 2021<br><br>37 study sites (29 in Greece and eight in Italy)	**Patients:**<br>Screening: n=1,060<br>Patients: n=606<br>A: n=405<br>C: n=189<br>Excluded: n=454<br><br><u>Follow-up:</u><br>until hospital discharge<br><br>**Inclusion criteria:**<br>– adult patients of either sex<br>– for women, unwillingness to remain pregnant during the study period<br>– confirmed infection by SARS-CoV-2 by molecular test<br>– findings in chest X-ray or chest computed tomography compatible with lower respiratory tract infection<br>– need for hospitalization<br>– plasma suPAR ≥6 ng ml−1<br><br>**Exclusion criteria:**<br>– any stage IV malignancy	**Anakinra**<br>1x/day 100mg subcutaneously at a final volume of 0.67 ml for 7-10 days	**Placebo**<br>1x/day 0.67 ml of 0.9% sodium chloride	**Primary outcome:**<br><u>11-point WHO-CPS at day 28, n (%)</u><br>Fully recovered PCR−<br>A: 204 (50.4)<br>C: 50 (26.5)<br>Asymptomatic PCR+<br>A: 40 (9.9)<br>C: 6 (3.2)<br>Symptomatic independent<br>A: 93 (23.0)<br>C: 74 (39.2)<br>Symptomatic assistance needed<br>A: 25 (6.2)<br>C. 21 (11.1)<br>Hospitalized with no need for oxygen<br>A: 9 (2.2)<br>C: 3 (1.6)<br>Hospitalized with nasal/mask oxygen<br>A: 8 (2.0)	"In conclusion, the SAVE-MORE trial showed that early start of treatment with anakinra guided by suPAR levels in patients hospitalized with moderate and severe COVID-19 significantly reduced the risk of worse clinical outcome at day 28."<br><br>**Comments:**<br><u>Limitations:</u><br>– the lack of enrollment of patients with critical COVID-19<br>– the difficulty for application of suPAR in all hospital settings<br>– inclusion of patients with SuPAR ≥ 6 ng ml^{-1}<br><br><u>Power-analysis:</u><br>"To replicate this primary effect size in the SAVE-MORE trial, and with a 90% power at the 5% significance level, a sample size of 200 was needed for the

Supplementary Information No 4 Characteristics of included studies

Declercq 2021
Declercq J, van Damme KFA, De Leeuw E, et al: Effect of anti-interleukin drugs in patients with COVID-19 and signs of cytokine release syndrome (COV-AID): a factorial, randomised, controlled trial. *Lancet Respir Med* 2021; 9:1427–38

Design	Study period and place	Participants	Intervention	Control	Outcomes	Conclusion
		− any do-not-resuscitate order<br>− ratio or partial oxygen pressure to fraction of inspired oxygen less than 150 mmHg<br>− need of NIV (CPAP or BPAP) or MV<br>− any primary immunodeficiency<br>− fewer than 1,500 neutrophils per mm3<br>− oral or intravenous intake of corticosteroids at a daily dose greater than or equal to 0.4 mg kg−1 of prednisone for a period longer than the last 15 d<br>− any anti-cytokine biological treatment, including JAK inhibitors, during the last 1 month<br>− severe hepatic failure<br>− end-stage renal failure necessitating hemofiltration or peritoneal hemodialysis<br>− pregnancy or lactation.<br><br>**Demographics (I/B1/B2):**<br>Age, mean (SD)<br>A: 62 (11.4)<br>C: 61.5 (11.3)<br>Male, n (%)<br>A: 236 (58.3)<br>C: 108 (57.1)<br><br>**Severity of condition according to respiratory support (n/N (%)):**<br>no supplemental oxygen:<br>A:39/405 (10); C:11/189 (6)<br>low or high flow supplemental oxygen:<br>A: 366/405 (90); C: 178/189 (94)<br>suPAR ≥ 6 ng/ml			C: 10 (5.3)<br>Need for HFO or NIV<br>A: 1 (0.2)<br>C: 1 (0.5)<br>MV with P/F > 150 mmHg<br>A: 1 (0.2)<br>C: 1 (0.5)<br>MV with P/F < 150 mmHg or vasopressors<br>A: 5 (1.2)<br>C: 4 (2.1)<br>MV with P/F < 150 mmHg and vasopressors or hemodialysis or ECMO<br>A: 6 (1.5)<br>C: 6 (3.2)<br>Dead<br>A: 13 (3.2)<br>C: 13 (6.9)<br><br>**Secondary outcomes:**<br>Median time to hospital discharge, d (IQR)<br>A: 11 (7.8)<br>C: 12 (8.5)<br>P = 0.033<br>Median time of ICU stay, d (IQR)<br>A: 10 (21)<br>C: 14 (22)<br>P = 0.026<br>At least one serious TEAE, *n* (%)<br>A: 65 (16.0)<br>C: 41 (21.7)<br>P = 0.107<br>At least one non-serious TEAE, n (%)<br>A: 335 (82.7)<br>C: 156 (82.5)<br>P = 1.00	placebo treatment arm and 400 for the anakinra treatment arm.”

Tharaux 2021

Supplementary Information No 4 Characteristics of included studies

Declercq 2021
Declercq J, van Damme KFA, De Leeuw E, et al: Effect of anti-interleukin drugs in patients with COVID-19 and signs of cytokine release syndrome (COV-AID): a factorial, randomised, controlled trial. *Lancet Respir Med* 2021; 9:1427–38

Design	Study period and place	Participants	Intervention	Control	Outcomes	Conclusion

The CORIMUNO-19 Collaborative Group: Effect of anakinra versus usual care in adults in hospital with COVID-19 and mild-to-moderate pneumonia (CORIMUNO-ANA-1): a randomised controlled trial. Lancet Respir Med 2021; 9:295-304

Design	Study period and place	Participants	Intervention	Control	Outcomes	Conclusion
Randomized controlled trial, open-label, Inpatient	08.04 – 26.04.2020<br><br>16 University hospitals in France	**Patients:**<br>Screening: n = 153<br>Patients: n = 116<br>A: n = 59<br>C: n = 57<br>Excluded: n=37<br>Follow-up:<br>14d for primary outcome and 28d or 90d for secondary outcomes<br><br>**Inclusion criteria:**<br>- adult patients<br>-confirmed SARS-CoV-2 infection (positive on real-time RT-PCR or chest CT scan typical of COVID-19 pneumonia, or both) with mild-to-moderate, severe, or critical pneumonia (ie, receiving oxygen at a flow of >3 L/min via mask or nasal cannula and a score of ≥5 points on the WHO Clinical Progression Scale [WHO-CPS] 10-point ordinal Scale<br>-C-reactive protein serum concentration of more than 25 mg/L not requiring admission to the hospital intensive care unit at the time of admission<br>-mild-to-moderate COVID-19 pneumonia with a WHO-CPS score of 5 points, receiving at least 3 L/min of oxygen but without ventilation assistance (eg, high-flow oxygen, non-invasive ventilation, or mechanical ventilation).<br><br>**Exclusion criteria:**<br>- known hypersensitivity to Anakinra or any of its excipients<br>- pregnancy<br>- current documented bacterial infection<br>- an absolute neutrophil count of 1·0 × 10^9 per L or less	**Anakinra**<br>2 x 200 mg (i.v.) daily on days 1–3, followed by 2 x 100 mg (i.v.) daily on day 4 and 100 mg (i.v.) /daily on day 5<br><br>In the absence of improvement (reduction in oxygen requirement by >50%) after 3 days, decision by practitioner:<br><br>2x 200 mg(i.v.) daily d4-6, then<br><br>2x 100 mg (i.v.) d7, then<br><br>1x 100 mg (i.v.) d8	**Standard of Care:**<br>Antibiotics, antiviral meds, corticosteroids, vasopressors, anticoagulants (practitioner's choice)	**Co-primary outcomes:**<br>The proportion of patients who had died or needed non-invasive or mechanical ventilation by day 4 (ie, a score of >5 on the WHO-CPS)<br><br>A: 21/59 (36%)<br>C: 21/55 (38%)<br><br>Survival without need for mechanical or non-invasive ventilation (including high-flow oxygen) at day 14.<br>median posterior HR: 0·97 (90% CrI 0·62 to 1·52)<br><br>Non-invasive ventilation, mechanical ventilation or death up to day 14<br>A: 28 (47%; 95% CI 33 to 59)<br>C: 28 (51%; 95% CI 36 to 62)<br><br>Mortality, at day 90<br>A: 16 (27%)<br>C: 15 (27%)<br><br>Serious adverse events, n (%), p<br>Patients with at least one serious adverse event<br>27 (46%) / 21 (38%), 0.45<br>Patients with multiple serious adverse events<br>8 (14%) / 5 (9%),	"Anakinra did not improve outcomes in patients with mild-to-moderate COVID-19 pneumonia. Further studies are needed to assess the efficacy of anakinra in other selected groups of patients with more severe COVID-19."<br><br>**Comments:**<br>Limitations<br>− No blinding<br>− usual care could differ among centres and over time<br>− small sample size<br>− wide CrIs and CIs<br>− no provision of an accurate measure of the ratio of partial pressure of oxygen to fractional concentration of oxygen in inspired air, because arterial blood gas measurements were not done<br>− narrow segment of the COVID-19 patient population targeted (patients with a WHO-CPS score of exactly 5 points and requiring at least 3 L/min of oxygen without any ventilatory support regardless of inflammatory status)

Supplementary Information No 4 Characteristics of included studies

**Declercq 2021**<br>Declercq J, van Damme KFA, De Leeuw E, et al: Effect of anti-interleukin drugs in patients with COVID-19 and signs of cytokine release syndrome (COV-AID): a factorial, randomised, controlled trial. *Lancet Respir Med* 2021; 9:1427–38						
**Design**	**Study period and place**	**Participants**	**Intervention**	**Control**	**Outcomes**	**Conclusion**
		- a platelet concentration<br>of less than 50 G/L<br>- serum aspartate aminotransferase or serum alanine aminotransferase of more than fivetimes the upper limit of normal<br>- severe renal insufficiency defined by an estimated glomerular filtration rate of less than 30 mL/min.<br><br>**Demographics:**<br><u>Age, median (IQR):</u><br>66 (59 - 76)<br>A: 67 (55.5 –74.3)<br>C: 64,9 (59.5 –78.3)<br><br><u>Male, n</u><br>80 (70%)<br><br>**Severity of condition according to respiratory support (n/N (%)):**<br>Low flow supplemental oxygen<br>A: 59/59 (100)<br>C: 55/55 (100)			**Secondary outcomes:**<br>- clinical status assessed with the WHO-CPS at days 4, 7, and 14;<br><br><u>Overall survival at days 14, 28, and 90</u><br>Mortality at day 14<br>A: 9 (15%)<br>C: 13 (24%)<br>Adjusted HR: 0·56 (95% CI 0·23 to 1·39)<br><br>Mortality at day 28<br>A: 13 (22%)<br>C: 13 (24%)<br>Adjusted HR: 0·77 (95% CI 0·33 to 1·77)<br><br>Mortality at day 90<br>A: 16 (27%)<br>C: 15 (27%)<br>Adjusted HR: 0·97 (95% CI 0·46 to 2·04)<br><br><u>time to discharge at day 28</u><br>A: 34 (58%)<br>C: 34 (62%)<br>Adjusted HR: 0·91 (95% CI 0·56 to 1·48)<br><br><u>Time to oxygen supply independency at day 28, n (%), adj. HR (95% CI)</u><br>37 (63%) / 38 (69%), 1.01 (95% CI 0.64 to 1.61)<br><br><u>time to negative viral excretion</u><br>not assessed due to paucity of data	

Supplementary Information No 4 Characteristics of included studies

**Declercq 2021**<br>Declercq J, van Damme KFA, De Leeuw E, et al: Effect of anti-interleukin drugs in patients with COVID-19 and signs of cytokine release syndrome (COV-AID): a factorial, randomised, controlled trial. *Lancet Respir Med* 2021; 9:1427–38						
**Design**	**Study period and place**	**Participants**	**Intervention**	**Control**	**Outcomes**	**Conclusion**
					biological factors (eg, C-reactive protein concentration<br><br>Adverse events, n (%), p<br>Patients with at least one adverse event<br>29 (49%) / 23 (42%), 0.46<br>Patients with multiple adverse events<br>19 (32%) / 14 (25%)<br><br>**Subgroup analysis:**<br>prespecified subgroup analysis according to antiviral drug use at baseline: too few patients were on antivirals at baseline to enable this analysis.	
