# Supplementary material for: Anakinra for the treatment of COVID-19 patients: a systematic review and meta-analysis
Source: Eur J Med Res. 2023 Feb 25;28:100. doi: 10.1186/s40001-023-01072-z (PMC9959952; doi:10.1186/s40001-023-01072-z)
Supplement: Supplementary file 5 — Additional file 5. Risk of bias assessment. [file 40001_2023_1072_MOESM5_ESM.pdf]

Supplementary Information No 5 Risk of bias assessment

Table 1 Risk of bias analysis for 28-day mortality

**Outcome**	**Study**	**Risk of Bias Domain**					
		**Randomisation process**	**Deviations from intended interventions**	**Missing outcome data**	**Measurement of the outcome**	**Selection of the reported results**	**Overall**
28-day mortality	Declercq 2021	Low	Low	Low	Low	Low	**Low**
	Derde 2021	Low	Low	Low	Low	Low	**Low**
	Kyriazopoulou 2021	Some concerns due to baseline differences	Low	Low	Low	Low	**Some concerns**
	Tharaux 2021	Low	Low	Low	Low	Low	**Low**

Table 2 Risk of bias analysis for 60-day mortality

Outcome	Study	Risk of Bias Domain					
		**Randomisation process**	**Deviations from intended interventions**	**Missing outcome data**	**Measurement of the outcome**	**Selection of the reported results**	**Overall**
60-day mortality	Declercq 2021	Low	Low	Low	Low	Low	**Low**

Table 3 Risk of bias analysis for mortality time-to-event at day 28

Outcome	Study	Risk of Bias Domain					
		**Randomisation process**	**Deviations from intended interventions**	**Missing outcome data**	**Measurement of the outcome**	**Selection of the reported results**	**Overall**
Mortality time-to-event, 28 day	Tharaux 2021	Low	Low	Low	Low	Low	**Low**
	Kyriazopoulou 2021	Some concerns due to baseline differences	Low	Low	Low	Low	**Some concerns**

Table 4 Risk of bias analysis for mortality time-to-event at day 90

Outcome	Study	Risk of Bias					
		**Randomisation process**	**Deviations from intended interventions**	**Missing outcome data**	**Measurement of the outcome**	**Selection of the reported results**	**Overall**
Mortality time-to-event, 90 day	Tharaux 2021	Low	Low	Low	Low	Low	**Low**
	Derde 2021	Low	Low	Low	Low	Low	**Low**

Table 5 Risk of bias analysis for mortality at hospital discharge

Outcome	Study	Risk of Bias					
		**Randomisation process**	**Deviations from intended interventions**	**Missing outcome data**	**Measurement of the outcome**	**Selection of the reported results**	**Overall**
Mortality at hospital discharge	Derde 2021	Low	Low	Low	Low	Low	**Low**
	Declercq 2021	Low	Low	Low	Low	Low	**Low**

Table 6 Risk of bias analysis for clinical worsening: new need for invasive mechanical ventilation or death at day 28

**Outcome**	**Study**	**Risk of Bias Domain**					
		**Randomisation process**	**Deviations from intended interventions**	**Missing outcome data**	**Measurement of the outcome**	**Selection of the reported results**	**Overall**
Clinical worsening: new need for invasive mechanical ventilation	Declercq 2021	Low	Low	Low	Low	Low	**Low**
	Kyriazopoulou 2021	Some concerns due to baseline differences	Low	Low	Low	Low	**Some concerns**

Table 7 Risk of bias analysis for clinical improvement: patients discharged alive

Outcome	Study	Risk of Bias Domain					
		Randomisation process	Deviations from intended interventions	Missing outcome data	Measurement of the outcome	Selection of the reported results	Overall
Clinical improvement: patients discharged alive	Declercq 2021	Low	Low	Low	Low	Low	**Low**
	Kyriazopoulou 2021	Some concerns due to baseline differences	Low	Low	Low	Low	**Some concerns**
	Tharaux 2021	Low	Low	Low	Low	Low	**Low**

Table 8 Risk of bias analysis for serious adverse events

**Bias**							
**Outcome**	**Study**	**Randomisation process**	**Deviations from intended interventions**	**Missing outcome data**	**Measurement of the outcome**	**Selection of the reported results**	**Overall**
Serious adverse events	Declercq 2021	Low	Low	Low	Low	Low	**Low**
	Kyriazopoulou 2021	Some concerns due to baseline differences	Low	Low	Low	Low	**Some concerns**
	Tharaux 2021	Low	Low	Low	Low	Low	**Low**

Table 9 Risk of bias analysis for any adverse events

Outcome	Study	Risk of Bias Domain					
		**Randomisation process**	**Deviations from intended interventions**	**Missing outcome data**	**Measurement of the outcome**	**Selection of the reported results**	**Overall**
Adverse events, any	Declercq 2021	Low	Low	Low	Some concerns due to lack of blinding	Low	**Some concerns**
	Tharaux 2021	Low	Low	Low	Some concerns due to lack of blinding	Low	**Some concerns**

Table 10 Risk of bias analysis for adverse events, grade 3-4

**Outcome**	**Study**	**Risk of Bias Domain**					
		**Randomisation process**	**Deviations from intended interventions**	**Missing outcome data**	**Measurement of the outcome**	**Selection of the reported results**	**Overall**
Adverse events, grade 3-4	Declercq 2021	Low	Low	Low	Some concerns due to lack of blinding	Low	**Some concerns**
