# Supplementary material for: Anakinra for the treatment of COVID-19 patients: a systematic review and meta-analysis
Source: Eur J Med Res. 2023 Feb 25;28:100. doi: 10.1186/s40001-023-01072-z (PMC9959952; doi:10.1186/s40001-023-01072-z)
Supplement: Supplementary file 6 — Additional file 6. Meta-analyses of Anakinra versus control. [file 40001_2023_1072_MOESM6_ESM.pdf]

Supplementary Information No 6 Meta-analyses of Anakinra versus control

Study or Subgroup	Anakinra Events	Anakinra Total	Control Events	Control Total	Weight	Risk Ratio M-H, Random, 95% CI
Declercq 2021	10	43	9	72	100.0%	1.86 [0.82, 4.21]
**Total (95% CI)**		**43**		**72**	**100.0%**	**1.86 [0.82, 4.21]**
Total events	10		9			

Heterogeneity: Not applicable
Test for overall effect: Z = 1.49 (P = 0.14)

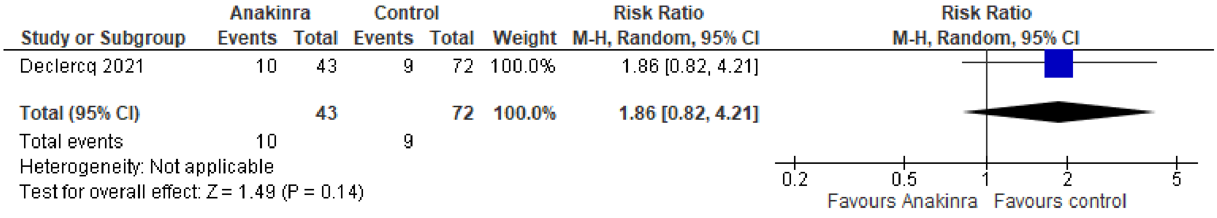

Fig. 1 Forest plot describing the difference between Anakinra compared to placebo or standard care alone regarding 60-day mortality. M-H = Mantel-Haenszel.

Study or Subgroup	log[Hazard Ratio]	SE	Anakinra Total	Control Total	Weight	Hazard Ratio IV, Random, 95% CI
Kyriazopoulou 2021	-0.7985	0.3889	405	189	55.3%	0.45 [0.21, 0.96]
Tharaux 2021	-0.2614	0.4323	59	55	44.7%	0.77 [0.33, 1.80]
**Total (95% CI)**			**464**	**244**	**100.0%**	**0.57 [0.32, 1.01]**

Heterogeneity: Tau² = 0.00; Chi² = 0.85, df = 1 (P = 0.36); I² = 0%
Test for overall effect: Z = 1.93 (P = 0.05)

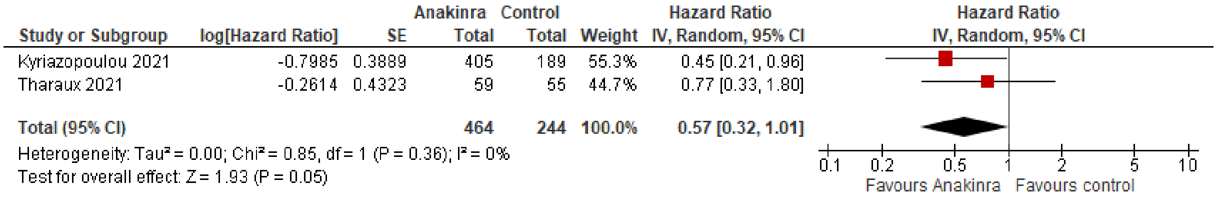

Fig. 2 Forest plot describing the difference between Anakinra compared to placebo or standard care alone regarding 28-day mortality, time-to-event. M-H = Mantel-Haenszel.

Study or Subgroup	log[Hazard Ratio]	SE	Anakinra Total	Control Total	Weight	Hazard Ratio IV, Random, 95% CI
Derde 2021	0	0	0	0		Not estimable
Tharaux 2021	-0.0305	0.3807	59	55	100.0%	0.97 [0.46, 2.05]
**Total (95% CI)**			**59**	**55**	**100.0%**	**0.97 [0.46, 2.05]**

Heterogeneity: Not applicable
Test for overall effect: Z = 0.08 (P = 0.94)

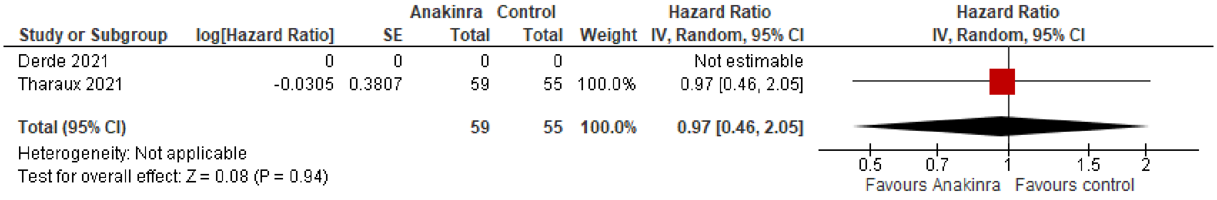

Fig. 3 Forest plot describing the difference between Anakinra compared to placebo or standard care alone regarding 90-day mortality, time-to-event. M-H = Mantel-Haenszel.

Study or Subgroup	Anakinra Events	Anakinra Total	Control Events	Control Total	Weight	Risk Ratio M-H, Random, 95% CI
Declercq 2021	10	44	9	74	22.9%	1.87 [0.82, 4.24]
Derde 2021	145	365	150	406	77.1%	1.08 [0.90, 1.29]
**Total (95% CI)**		**409**		**480**	**100.0%**	**1.22 [0.77, 1.92]**
Total events	155		159			

Heterogeneity: Tau² = 0.06; Chi² = 1.67, df = 1 (P = 0.20); I² = 40%
Test for overall effect: Z = 0.86 (P = 0.39)

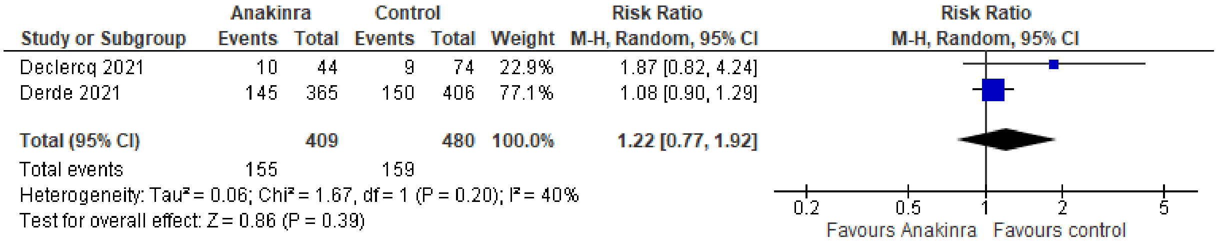

Fig. 4 Forest plot describing the difference between Anakinra compared to placebo or standard care alone regarding mortality at hospital discharge. M-H = Mantel-Haenszel.

Study or Subgroup	Anakinra Events	Anakinra Total	Control Events	Control Total	Weight	Risk Ratio M-H, Random, 95% CI
Kyriazopoulou 2021	25	405	24	189	100.0%	0.49 [0.29, 0.83]
**Total (95% CI)**		**405**		**189**	**100.0%**	**0.49 [0.29, 0.83]**
Total events	25		24			

Heterogeneity: Not applicable
Test for overall effect: Z = 2.65 (P = 0.008)

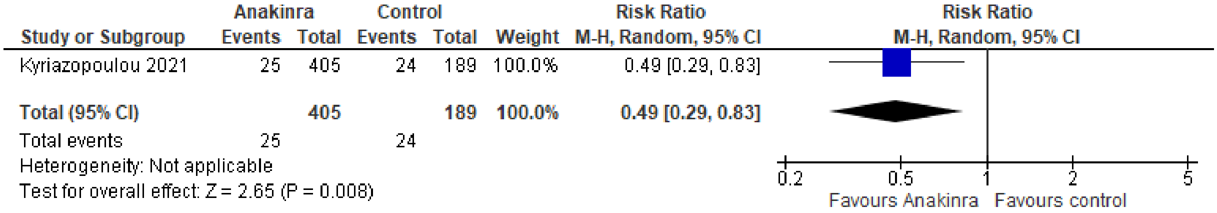

Fig. 5 Forest plot describing the difference between Anakinra compared to placebo or standard care alone regarding worsening of clinical status in patients with suPAR ≥ 6 ng/ml. M-H = Mantel-Haenszel.

Study or Subgroup	Anakinra Events	Anakinra Total	Control Events	Control Total	Weight	Risk Ratio M-H, Random, 95% CI
Declercq 2021	12	43	14	72	22.7%	1.44 [0.73, 2.81]
Kyriazopoulou 2021	65	405	41	189	41.7%	0.74 [0.52, 1.05]
Tharaux 2021	27	59	21	55	35.6%	1.20 [0.77, 1.85]
**Total (95% CI)**		**507**		**316**	**100.0%**	**1.02 [0.68, 1.53]**
Total events	104		76			

Heterogeneity: Tau² = 0.07; Chi² = 4.52, df = 2 (P = 0.10); I² = 56%
Test for overall effect: Z = 0.10 (P = 0.92)

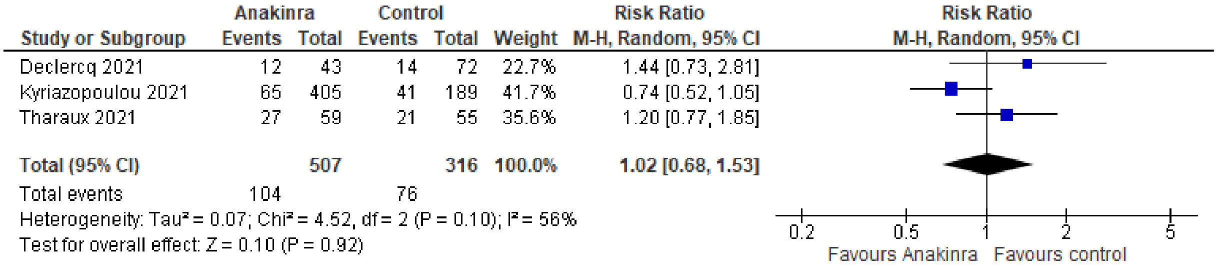

Fig. 6 Forest plot describing the difference between Anakinra compared to placebo or standard care alone regarding serious adverse events. M-H = Mantel-Haenszel.

Study or Subgroup	Anakinra Events	Anakinra Total	Control Events	Control Total	Weight	Risk Ratio M-H, Random, 95% CI
Declercq 2021	26	43	43	72	63.5%	1.01 [0.74, 1.38]
Tharaux 2021	29	59	23	55	36.5%	1.18 [0.78, 1.76]
**Total (95% CI)**		**102**		**127**	**100.0%**	**1.07 [0.84, 1.37]**
Total events	55		66			

Heterogeneity: Tau² = 0.00; Chi² = 0.34, df = 1 (P = 0.56); I² = 0%
Test for overall effect: Z = 0.53 (P = 0.59)

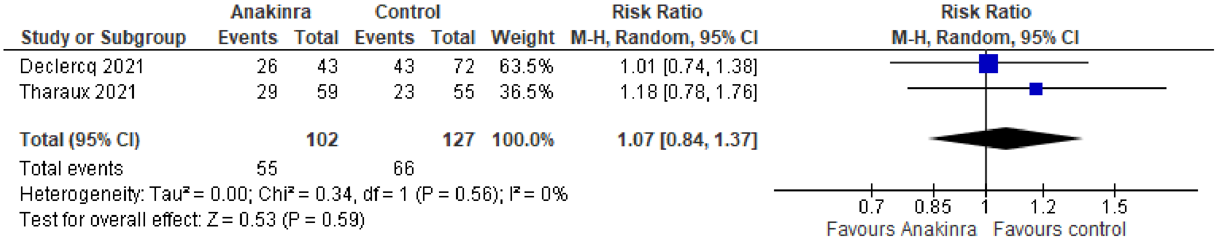

Fig. 7 Forest plot describing the difference between Anakinra compared to placebo or standard care alone regarding any adverse events. M-H = Mantel-Haenszel.

Study or Subgroup	Anakinra Events	Anakinra Total	Control Events	Control Total	Weight	Risk Ratio M-H, Random, 95% CI
Declercq 2021	16	43	24	72	100.0%	1.12 [0.67, 1.85]
**Total (95% CI)**		**43**		**72**	**100.0%**	**1.12 [0.67, 1.85]**
Total events	16		24			

Heterogeneity: Not applicable
Test for overall effect: Z = 0.42 (P = 0.67)

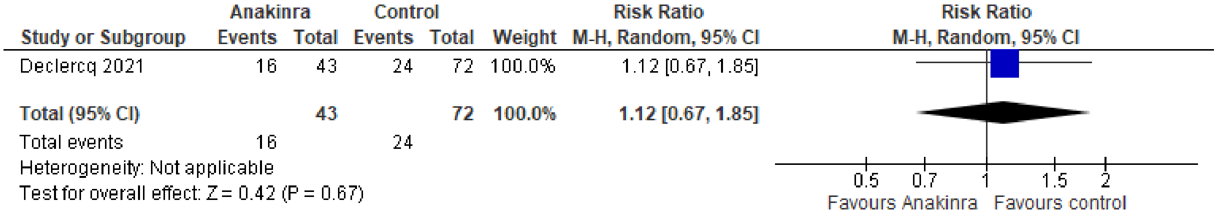

Fig. 8 Forest plot describing the difference between Anakinra compared to placebo or standard care alone regarding adverse events, grade 3-4. M-H = Mantel-Haenszel.
